# Nutrient intakes related to osteoporotic fractures in men and women – The *Brazilian Osteoporosis Study *(BRAZOS)

**DOI:** 10.1186/1475-2891-8-6

**Published:** 2009-01-29

**Authors:** Marcelo M Pinheiro, Natielen J Schuch, Patrícia S Genaro, Rozana M Ciconelli, Marcos B Ferraz, Lígia A Martini

**Affiliations:** 1 Rheumatology Division, Universidade Federal de São Paulo/EPM, São Paulo, Brazil; 2 Department of Nutrition, Faculdade de Saúde Pública, Universidade de São Paulo, São Paulo, Brazil; 3 São Paulo Health Care Economics Center, Universidade Federal de São Paulo/EPM, São Paulo, Brazil; 4 Av. Dr. Altino Arantes, 669, apto 105, Vila Clementino, São Paulo, SP, Brazil

## Abstract

**Background:**

Adequate nutrition plays an important role in bone mass accrual and maintenance and has been demonstrated as a significant tool for the prevention of fractures in individuals with osteoporosis.

**Objective:**

The aim of the present study was to evaluate bone health-related nutrients intake and its association with osteoporotic fractures in a representative sample of 2344 individuals aged 40 years or older in Brazil.

**Methods:**

In a transversal population-based study, a total of 2420 individuals over 40 years old were evaluated from March to April 2006. Participants were men and women from all socio-economic classes and education levels living around the Brazilian territory Individuals responded a questionnaire including self reported fractures as well a 24-hour food recall. Nutrient intakes were evaluated by Nutrition Data System for Research software (NDSR, University of Minnesota, 2007). Low trauma fracture was defined as that resulting of a fall from standing height or less. Nutrient intakes adequacies were performed by using the DRI's proposed values. Statistical analysis comprises Oneway ANCOVA adjusted by age and use of nutritional supplements and multiple logistic regression. SAS software was used for statistical analysis.

**Results:**

Fractures was reported by 13% of men and 15% of women. Women with fractures presented significantly higher calcium, phosphorus and magnesium intakes. However, in all regions and socio-economical levels mean intakes of bone related nutrients were below the recommended levels. It was demonstrated that for every 100 mg/phosphorus increase the risk of fractures by 9% (OR 1.09; IC95% 1.05–1.13, p < 0.001).

**Conclusion:**

The results demonstrated inadequacies in bone related nutrients in our population as well that an increase in phosphorus intake is related to bone fractures.

## Background

Adequate nutrition plays an important role in bone mass accrual and maintenance and has been demonstrated as a significant tool for the prevention of fractures in individuals with osteoporosis. Not only calcium but also protein, phosphorus, magnesium, vitamin D and K intakes are important factors related to bone health [1,2].

In adults, low calcium and vitamin D intakes may negatively impact on bone mass, trough increased PTH secretion with consequent mobilization of calcium from the skeleton to the blood stream aiming to maintain its critical biological functions and mineral homeostasis. Moreover, several clinical trials have demonstrated that calcium supplementation can minimize secondary hyperparathyroidism, bone loss and the risk of fragility fractures [3,5].

The relation between vitamin D and bone health has been well established. Vitamin D deficiency or abnormalities in its metabolism are associated with lower intestinal calcium absorption, increased PTH secretion and increased bone resorption [6]. Until recently it was believed that vitamin D deficiency was uncommon in equatorial areas and lower latitude regions. However, several studies in the past decade have shown that vitamin D deficiency is broadly common and affects all the different continents, age groups and socio-economic classes [7,9].

Magnesium is essential for the normal function of the parathyroid glands, vitamin D metabolism, and adequate sensitivity of target tissues to PTH and active vitamin D metabolite. There is some evidence showing that magnesium supplementation in postmenopausal women seems to increase bone mineral density (BMD). On the other hand, there is no solid data on its effect on the reduction of fracture risk [10].

Recent studies have demonstrated that patients with osteoporosis have increased levels of biochemical markers of vitamin K deficiency [11]. Such markers are associated with higher risk of fractures. It also seems that moderate supplementation doses of vitamin K are associated with better bone status [12].

Excessive intake of certain nutrients, such as protein, phosphorus and vitamin A, may have a negative impact on bone and mineral metabolism. Elevated protein and phosphorus intake is associated with higher renal loss of calcium, increased PTH secretion and higher bone resorption of calcium, phosphorus and magnesium [13,14]. Adequate intake of such nutrients has a fundamental role for bone homeostasis maintenance. Conversely, low protein intake is also deleterious for bone mass since protein intake is critical for the production and secretion of growth factors, especially growth hormone and insulin-like growth factor I (IGF-I), and for the synthesis of type I collagen and many other non-collagen proteins in the bone matrix (osteocalcin, bone sialoprotein and matrix Gla protein). It has been demonstrated that protein supplementation in undernourished elderly can increase IGF-I production [14]. IGF-I effects on bone mass seem to be related to a stimulation of osteoblasts recruitment and differentiation. On the other hand, excessive vitamin A intake, especially through the use of supplements, is associated with higher bone resorption, bone formation inhibition and higher risk of fractures [15,16].

Epidemiological studies that evaluate nutrient intakes in individuals with or without fractures are scarce in the Brazilian population. In the present study, we investigate nutrient intake and its association with self-reported osteoporotic fractures in a representative sample of the Brazilian population over 40 years old.

## Patients and methods

### Subjects

In a transversal population-based study, a total of 2420 individuals over 40 years old were evaluated from March to April 2006. Participants were men and women from all socio-economic classes and education levels living around the Brazilian territory (five geographic regions, 150 municipalities). Individuals were invited to participate in a quantitative survey to characterize clinical risk factors for fragility fractures. A structured questionnaire was especially designed for the present study based on literature review. The main parameters evaluated were: age, demographic, anthropometric and socio-economical data, general knowledgement of osteoporosis, previous fall and circumstances of fall in the last year, medical history, previous fracture, gynecological and reproductive history, familial history of hip fracture after age 50 years in first degree relatives, quality of life (SF-8), medication use and co-morbidities classified according to the ICD, International Classification of Diseases, 10^th ^revision. The survey consisted of home-applied personal interviews conducted by trained investigators.

Sample size was calculated by probabilistic analysis to represent the Brazilian urban and rural population. Calculations and study design were based on data from the last National Census (Brazilian Institute of Geography and Statistics, IBGE, 2000) and the National Survey of Domicile Sampling (PNAD, 2003). Domiciles were randomly selected and interviews were performed in week days and weekends, day or night, from March to April 2006, in order to maximize the chance of finding the target-individuals at home. The data were later weighed to reconstruct the distribution and the proportionality originally observed for the total Brazilian population. Sampling error was ± 2.2% with 95% confidence intervals.

Socioeconomic status classification used in the present study reflects the individual and household purchasing power and takes into account a list of assets as well as the educational level of the head of household. As described, patients are classified as A, B, C, D or E depending on their score. A status has the highest purchasing power and E the lowest. For the purposes of this study, A is considered upper class, B middle class and C, D and E lower class.

Osteoporotic or low energy fracture was defined as those associated with fall from standing height or less after age 50 years. Skeletal sites for fragility fractures were axial (ribs, lumbar and thoracic vertebrae) and peripheral bones (forearm, humerus and femur). Traumatic fractures and those occurring at sites not characteristic of bone fragility (face, skull, tibia, fibula and femoral diaphisis) were excluded from the analysis. Individuals experiencing 2 or more falls in the last 12 months were defined as chronic fallers.

The presence of cognition deficiencies (neurological diseases or senile dementia) that could impair the participant to give consistent and trustable answers and the presence of more than two individuals over 40 years old in the domicile was considered exclusion criteria in the study.

### Food Intake

Food intake was assessed by using 24-hour recalls (24R). Upon personal interviews, participants reported in details all the food and beverages consumed the day before, starting from the first food ingested after wakening until the last meal before going to bed and including food taken inside and outside the domicile. The 24R was applied at home and filled by an interviewer trained in the method by an experienced nutritionist. Consistence assessment and corrections were made in the applied questionnaires in order to ensure the accuracy of the notations reported.

Measurements of food and recipes were standardized according to an in house table of home measures. Food data were converted in the respective values of macro and micronutrients and analyzed using the software *Nutrition Data System *for Research (NDS-R. University of Minnesota), version 2005.

For nutrient intake evaluation only complete 24R records with energy intake between 400 and 5500 kcal/day were considered. Results refer to a total of 2344 individuals (693 men and 1651 women).

Mean intake for each nutrient was compared to the proposed *Dietary Reference Intakes *(DRIs) from the Medicine Institute [2,17]., according to gender and age group. Briefly, daily recommendation for calcium (1000–1200 mg), vitamin D (5–15 μg), magnesium (350 mg for men and 265 mg for women), vitamin K (120 mg for men and 90 mg for women) and vitamin A (625 μg RAE for men and 500 μg RAE for women), total protein (56 g/day for men and 46 g/day for women) and phosphorus (780 mg/day for men and 580 mg/day for women) (DRIs, 1997–2001).

All the nutrients were adjusted for energy according to Willet & Stampfer method [18]. Energy intake from food was evaluated according to the recommendations from the Food and Agricultural Organization/WHO (2001) for gender, age group and physical activity levels [19].

All questionnaires were revised by an independent supervisor and underwent continuous process of criticism and consistency. Inconsistently filled questionnaires were returned for correction. About 25% of the questionnaires were verified *in loco *or *post hoc *by phone call.

### Anthropometrics

Body weight (kg) was measured (after removal of shoes and heavy outer clothing) using a balance beam scale. Height (m) was measured (after removal of shoes) using a stadiometer. Height and body weight were used to calculate body mass index (BMI, kg/m^2^). Nutritional status was categorized according to WHO classification (1995) [20].

The study protocol was revised and approved by the UNIFESP/EPM's Ethics and Research Committee.

### Statistical Analysis

Variables distribution was assessed by Kolmogorov-Smirnov's test. For comparison between male and female population, Mann-Whitney U test was used. Qui-square analysis was performed for categorical variables between individuals with and without fractures. To analyze differences between the groups among gender and country regions, nutrients were adjusted for age and use of nutritional supplements and the Oneway ANCOVA test was performed.

In order to investigate the risk factors for fractures, the multiple logistic regression analysis was performed considering the presence of fractures as dependent variable. Nutrient intakes, age, weight, height and use of nutritional supplements were considered independent variables. All analysis were performed using the software SAS, version 8.02 (SAS Institute Inc, 1999–2001, Cary, NC, USA). Significance level was set as p < 0.05.

## Results

Characteristics of the evaluated population are shown in Table 1. Men had weight and height significantly higher than women, while BMI did not differ between genders. According to FAO/WHO classification, men and women, on average, are pre-obese. About 57% of the individuals had BMI higher than 25 kg/m^2^.

**Table 1:** Characteristics of Brazilian population according to gender.

	**Men (n = 693)**	**Women (n = 1651)**	**Total (n = 2344)**
**Age (years)**	56 (40–98)^a^	59 (40–102) *	58 (40–102)
**Body Mass Index (kg/m**^2^**)**	25.8 (14.7–46.1)	25.7 (12.7–51.2)	25.7 (12.7–51.2)
**Weight (kg)**	72.0 (40–130)	63.7 (30–125) *	66 (30–130)
**Height (m)**	1.66 (1.40–2.00)	1.57 (1.20–1.83) *	1.60 (1.20–2.00)

^a ^Median (minimum-maximum) *p < 0.001 men vs. women, Mann-Whitney Test.

Fragility fractures in Brazilian population is demonstrated in Table 2. Men and women had similar percentage of fractures, particularly in people with more than 70 years old (p < 0.05). As expected, fractures were significantly higher in people with osteoporosis (25% vs. 13%, osteoporosis vs. non osteoporosis respectively, p = 0.001).

**Table 2:** Presence of fragility fracture in Brazilian population, according to gender, age, nutritional supplements use and presence of osteoporosis.

		**Fragility Fractures**	
		**No**	**Yes**
**Gender**	Male	603 (87%)	89 (13%)
	Female	1353 (84%)	241 (15%)
**Age (years)**	40 – 49	638 (88%)	88 (12%)
	50 – 59	397 (88%)	56 (12%)
	60 – 69	359 (85%)	62 (15%)
	70 – 79	411 (82%)	88 (18%) *
	≥ 80	153 (81%)	36 (19%) *
**Osteoporosis**	No	1809 (87%)	280 (13%)
	Yes	150 (75%)	50 (25%) *

* p < 0.001

Energy and bone health-related nutrients intakes according to osteoporotic fractures and gender are shown in Table 3. Overall recommended energy intake from the diet for men and women are 1710 and 1240 kcal/day, respectively. In our population, men had energy intake lower than the recommended (about 100 kcal/day less) while women had values very close to the recommended amount. No significant differences regarding energy intake was observed between male and female with or without fractures.

**Table 3:** Energy and micronutrient intakes in Brazilian population, according to presence of fractures and gender.

	**Fragility Fractures**			
**Daily Intake**	**No**		**Yes**	
	**Male**	**Female**	**Male**	**Female**
**Energy (kcal)**	1331^a^ (610 – 3098)	1197 (427 – 3565)	1199 (405–5128)	1331 (610–3098)
**Protein (g)**	62 (51–73)	59 (49–68)	66 (50–76)	60 (53–71)
**Calcium (mg)**	359 (255–503)	372 (276–518)	382 (263–545)	414* (296–591)
**Phosphorus (mg)**	737 (632–871)	730 (640–848)	760 (665–882)	772* (662–934)
**Magnesium (mg)**	201 (161–245)	189 (158–223)	244 (159–244)	196* (164–235)
**Vitamin D (μg)**	1.8 (0.8–2.9)	1.9 (1.1–3.2)	1.6 (0.9–3.1)	2.2 (1.2–3.6)
**Vitamin K (μg)**	41 (29–64)	43 (34–74)	42 (29–75)	39 (31–71)
**Vitamin A (μg REA)**	131 (18–265)	138 (68–257)	212 (116–335)	231 (141–376)

^a ^median (minimum-maximun). REA: retinol equivalent activity. *Oneway ANCOVA, adjusted for age and use of nutritional supplements, p < 0.05 male vs female with fracture

Nutrients intake was adjusted for energy in order to determine the net intake of the nutrient without the effect of the energy. In this way, one can minimize higher nutrients intakes related to high energy dietary intake. After adjustments for energy, we observed significant differences between genders in terms of calcium, phosphorus and magnesium intakes. Women with fractures presented significantly higher calcium, phosphorus and magnesium intakes compared to females without fractures. No significant difference was observed between men with or without fractures.

According to the DRIs daily recommendations for calcium, vitamin D, magnesium, vitamin K and vitamin A, mean intake of our population with or without fractures was below the recommended amounts. Total protein and phosphorus intakes were close to the recommended quantities.

Energy-adjusted intakes of nutrients for the population according to the geographic regions in Brazil are shown in Table 4. No significant difference was observed for vitamin K intake among the five geographic regions. However, significant differences were observed for protein, calcium, phosphorus, magnesium, vitamin D and A intakes among the different regions. People with fractures living in the North region had higher intakes of calcium, phosphorus, magnesium and vitamin D. Individuals living in the Northeast presented higher protein intake while the highest vitamin A intake was seen for Central residents.

**Table 4:** Micronutrient intakes in Brazilian population, according to country regions and presence of fractures.

	**South**		**Southeast**		**Central**		**Northeast**		**North**	
**Fractures**	**No**	**Yes**	**No**	**Yes**	**No**	**Yes**	**No**	**Yes**	**No**	**Yes**
**Micronutrient**										
**Protein (g/d)**	60^a^ 52–71	61 53–73	59 49–69	60 53–72	59 50–70	61 53–75	58 48–69	64* 55–70	62 50–71	60 50–83
**Calcium (mg/d)**	396 288–565	410 303–585	387 294–513	396 290–555	364 271–513	473* 340–669	339 250–518	371 250–511	325 239–481	454* 310–678
**Phosphorus (mg/d)**	787 659–869	754 665–831	730 645–848	765 653–900	725 645–823	863* 695–998	721 612–878	742 662–882	741 611–877	821* 670–1089
**Magnesium (mg/d)**	182 160–219	186 164–210	198 167–230	191 163–223	197 163–242	209 175–252	190 150–234	193 153–230	178 138–236	213* 164–281
**Vitamin D (μg/d)**	2.1 1.1–3.3	2.2 1.3–3.6	1.8 0.0–2.8	1.8 0.7–3.0	1.7 0.9–2.8	2.2 1.1–4.0	1.9 1.0–3.2	1.8 1.1–3.0	2.1 1.1–3.5	3.6* 1.4–5.3
**Vitamin K (μg/d)**	46 35–101	43 36–80	47 36–83	44 35–81	45 33–69	40 32–73	35 24–46	33 27–44	36 26–55	37 24–72
**Vitamin A (μg REA/d)**	254 142–375	250 168–368	198 116–338	199 128–361	160 43–284	242* 155–354	167 56–307	169 85–272	159 67–264	188 82–399

^a ^median (minium-maximun) * p < 0.05 Oneway ANCOVA whitin groups, adjusted for age and nutritional supplement. REA: retinol equivalent activity.

Individuals in the higher classes (A and B) did not present significantly higher nutrient intakes compared to individuals in the lower socio-economic classes (C, D and E). However, individuals in the C, D and E classes with fractures presented significantly higher phosphorus intakes compared to individuals at same class without fractures (Class C: 774 vs 740 mg/d; Class D+E: 756 vs 722 mg/d, with vs without fractures respectively, p < 0.05).

In the logistic analysis for fractures, age higher than 60 years old and higher phosphorus intake were considered as relevant risk factors. Individuals with more than 60 years old had 45% more risk of fracture and for every 100 mg/phosphorus intake there is a 9% increase the risk of fracture (Table 5).

**Table 5:** Final multiple logistic regression model for fragility fractures in Brazilian men and women.

**Variable**		**P value**	**Odds Ratio**	**IC 95%**
**Age**	< 60 years	---	---	---
	≥ 60 years	0.002	1.45	1.14 – 1.83
**Phosphorus intake**	Every 100 mg	<0.001	1.09	1.04 – 1.13

## Discussion

In the present study we detected important inadequacies for the intake of bone health-related nutrients as calcium and vitamin D in the Brazilian population. Furthermore, for every 100 mg of phosphorus intake the risk of fractures increases by 9%.

The potential adverse effects of higher phosphorus intake on bone metabolism has been investigated by several authors in the last decades. It has been demonstrated that a high phosphorus diet produces hormonal changes of mild hyperparathyroidism, lowering calcitriol concentrations thus disrupting calcium homeostasis (13,14,21).

In 1996 Calvo & Park, reviewed the literature regarding the bone effects of high phosphorus diet in animals and humans. Studies with high phosphorus content in animals from different species, demonstrated increased bone resorption, reduced bone formation as well reduced bone mineral content. In humans such effect was not observed, even more the secondary hyperparathyroidism theory was also observed with a low calcium diet [3], leading to the assumption that the high phosphorus/low calcium diet was more prone to induce loss of bone mineral density.

However, the Food and Nutrition Board questioned the calcium/phosphorus concept. It has been demonstrated that the ingested ratio must consider the differing absorption efficiencies, mainly in elderly, when calcium absorption drops more sharply than does phosphorus absorption [2]. In fact, Heaney and Recker (1987) in a calcium kinetic study demonstrated that increasing phosphorus intake from 1.1 to 2.3 g showed no effect on bone turnover [22].

Additionally, studies investigating the relationship between nutrient intakes and bone metabolism, did not demonstrated that phosphorus *per se *induced bone loss (2, 3, 23). By the other hand, low calcium intake has been demonstrated to contribute to bone fracture.

Evaluating the effects of diet and fracture risk in a prospective study in 34.696 British women and men, Key *et al *2007, demonstrated that the fracture risk was significantly higher among women with a calcium intake lower than 525 mg/d [23].

Calcium plays a fundamental role in global and bone health and has recently received significant attention in terms of public health policies to ensure its adequate intake. Calcium intake lower than the recommended amounts required for good bone health has been observed in all age groups around the world, even in developed countries [5].

Calcium intake in our sample was, on average, about one third of the recommended amount for gender and age group. Alarmingly, about 99% of the population had calcium intake lower than the recommended (1200 mg/day). Similar results have also been observed in 20 to 60 years old individuals residing in the São Paulo state, Brazil, where the authors found a energy-adjusted intake of calcium of 448 mg/day [24].

Vitamin D deficiency is also associated with increased risk of osteoporosis and fragility fractures. The main source of 25(OH)D_3 _is exposure to sunlight which depends upon the latitude, season, skin pigmentation, gender, age, clothing and use of sunscreen. Dietary sources of vitamin D are scarce but have become especially relevant given the common current behavior of avoiding sunlight exposure to prevent skin cancer. Inadequate vitamin D intake has been reported in many populations. Mean vitamin D intake for men and women in the USA and UK are 8.12 and 7.33 μg, and 4.2 and 3.7 μg, respectively [25]. The higher vitamin D intake in the American population might be related to a strong policy for food fortification. Even with fortified food and with an intake mainly based on alimentary sources such as fish, Norwegian men and women have daily intake of vitamin D of only 6.8 and 5.9 μg, respectively. In Japan, the scenario is not much different and Japanese women have mean daily intake of vitamin D of 7.1 μg [26]. Some studies have shown that nutritional interventions directed to achieve adequate vitamin D intake can have a positive impact on bone health. Nakamura *et al *[26] have demonstrated that the consumption of fish (four times per week) was positively associated with 25(OH)D_3 _serum levels. Trivedi *et al *[27] in a prospective randomized double-blind placebo-controlled trial observed that quadrimestral supplementation with vitamin D 800 IU or 20 μg was associated with a 33% reduction in fracture risk in the elderly.

In BRAZOS, mean vitamin D intake was about one quarter of the recommended for gender and age group. Similarly to calcium intake, most of our sample (99.3%) had vitamin D intakes lower than the recommended amount. Given the pivotal roles these nutrients play on mineral homeostasis, we believe that urgent measures need to be taken in order to optimize bone health in our population. Simple and low cost changes would be helpful in this scenario: educational programs aiming to increase the intake of these nutrients from alimentary sources, use of fortified food and supplements and optimized exposure to sunlight whenever possible and safe.

Magnesium intake was also inadequate in our population. Mean magnesium intake was low yet close to the recommended amount. Only 20% of the participants reached the DRI for this nutrient. Similar results have been reported in NHANES III and also in the *Framingham Osteoporosis Study *[28]. In postmenopausal women, there is a positive significant association between magnesium intake and lumbar spine and femur BMD [29]. Magnesium intake has also been associated with BMD changes in pre-menopausal women receiving calcium supplementation [30]. It is important to point out that the observed inadequacy of magnesium intake in great part of our population does not necessarily translate into nutritional deficiency. Indeed, the assessment of magnesium nutritional status requires also measurements of its serum levels in order to better define inadequacy.

A similar approach is also needed to evaluate the results concerning vitamins A and K. Serum levels measurements are more sensitive and accurate to determine the presence of deficiency of these vitamins. In the BRAZOS population, the intake of vitamins A and K were below the recommendation for great part of the sample. Half of the individuals had vitamin A intakes below the recommended amount. Most of the participants (80%) also had vitamin K intakes under the recommendation for gender and age group.

The importance of adequate vitamin K intake and the maintenance of its serum levels on bone density and risk of fractures has been demonstrated in several studies. Lower vitamin K serum levels are associated with higher risk of fragility fractures [11,31]. Excessive vitamin A intake seems to be related to higher bone resorption and inhibition of bone formation with consequent higher risk of fractures [15,16]. In our population a small percentage of individuals had vitamin A intakes higher than the recommended. As vitamin A serum levels were not measured neither were bone markers assessed to evaluate potential toxic effects, we can not assure that those individuals are at higher risk for bone disease. Moreover, the higher vitamin A intake observed might be related to the ingestion of food rich in vitamin A only in the particular day of the interview.

In spite of the insufficient intake of most of the micronutrients studied, most of the population had adequate intake of protein. Similar results were reported in the *Framingham Osteoporosis Study*, where the authors observed a 30% inadequacy rate for protein intake [32]. Low protein intake has a negative effect on bone health and is associated with lower femur BMD measurements [13,32].

Some limitations of the present study need to be pointed out, especially regarding the method used to evaluate food intake (24-hour recall). One could not assure that food consumed at one day reflects accurately the usual intake of the individual and so our results must be interpreted with caution. To confirm nutritional inadequacy and properly address fortification/supplementation policies, dietary records including more days are needed as well as biochemical markers to access the nutrients bioavailability and their real adequacy. Besides, we did not perform bone mass measurements and serum biomarkers of bone metabolism. However, recently the fragility fractures are defined as the best outcome to evaluate bone health.

Other important point is that besides lower than recommended values, a significantly higher intake of calcium, phosphorus and magnesium was observed in women with fragility fractures. Such observation could indicate that improvement on dietary habits could minimize osteoporotic fractures in Brazilian population. However, we did not find association between nutrient intakes related to bone health and physical activity.

In summary, our results demonstrated that independently on the geographic region or the socio-economic status in Brazilian men and women an increase in phosphorus intake was a significantly related to bone fractures. Furthermore, a lower intake of calcium and vitamin D was observed in our population. Health professional dealing with this population ought to be aware of this situation and search for means of minimizing or neutralizing the negative effects of nutritional inadequacy on bone health.

## Competing interests

The authors declare that they have no competing interests.

## Authors' contributions

MMP and LAM: were responsible for the study design, statistical analysis e paper elaboration; NOJ: performed all the nutrient calculation and participated in paper elaboration; PSG: participated in the nutrient calculation an results discussion. RMC and MBF: was responsible for the study design and paper elaboration
